# Neuroprotective Effects of a Combination of Dietary Trans-Resveratrol and Hesperidin Against Methylglyoxal-Induced Neurotoxicity in a Depressive Amnesia Mouse Model

**DOI:** 10.3390/nu17091548

**Published:** 2025-04-30

**Authors:** Seon-Hyeok Kim, Seong-Min Hong, Eun-Ji Ko, Min-Jeong Park, Ji-Youn Kim, Sun-Yeou Kim

**Affiliations:** 1 College of Pharmacy and Institute of Pharmaceutical Science, Gachon University, Incheon 21936, Republic of Korea; ssun0701@gachon.ac.kr (S.-H.K.); hongsm0517@gmail.com (S.-M.H.); imko1004@gacho.ac.kr (E.-J.K.); alsendalsend99@naver.com (M.-J.P.); 2 Department of Exercise Rehabilitation, Gachon University, Incheon 21936, Republic of Korea

**Keywords:** *trans*-resveratrol, hesperidin, methylglyoxal, advanced glycation end-products, neurodegeneration, Alzheimer’s disease, depression, memory dysfunction, dietary supplement, neuroprotection

## Abstract

Background: Methylglyoxal (MGO), a reactive dicarbonyl compound, has been implicated in the formation of advanced glycation end-products (AGEs) and neuronal dysfunction. This study investigated the neuroprotective effects of the combination of trans-resveratrol and hesperidin (tRES-HESP) against MGO-induced neurotoxicity, focusing on memory dysfunction and depression-like behavior. Methods: Neuroblastoma 2a (N2a) cells were treated with MGO to induce neurotoxicity. The effects of tRES-HESP on cell viability, reactive oxygen species (ROS) production, apoptotic markers (BAX/Bcl 2 ratio, caspase 3 activity, and poly [ADP ribose] polymerase cleavage), and components of the glyoxalase system (glyoxalase-1, glyoxalase- 2, and receptors for AGEs) were assessed. The activation of the Kelch-like ECH-associated protein 1/Nuclear factor erythroid-2-related factor 2/Heme oxygenase-1 (Keap1/Nrf2/HO-1) pathway was also evaluated. In vivo, mice with MGO-induced depressive amnesia were treated with tRES-HESP (200 mg/kg) for eight weeks, and behavioral, biochemical, and histological assessments were performed. Results: tRES-HESP significantly reduced MGO-induced cytotoxicity, ROS production, and apoptosis in N2a cells. In addition, it restored the glyoxalase system and activated the Keap1/Nrf2/HO-1 pathway. In an in vivo model, tRES-HESP improved memory and depression-like behaviors, reduced cortisol and interleukin (IL)-6 levels, increased IL-10 levels, and lowered the expression of amyloid precursor protein and amyloid beta. Furthermore, tRES-HESP protected CA2/3 hippocampal subregions from MGO-induced damage. tRES-HESP exhibited neuroprotective effects through antioxidant, anti-apoptotic, and anti-inflammatory mechanisms. Conclusions: Our results suggest that tRES-HESP is a potential dietary supplement for preventing cognitive decline and depression, particularly in neurodegenerative conditions such as Alzheimer’s disease. Further studies are required to assess its clinical relevance and efficacy in the human population.

## 1. Introduction

Alzheimer’s disease (AD) is a neurodegenerative disorder that leads to memory impairment and also causes depression-like symptoms, posing significant challenges to public health [1]. Despite extensive research, current treatments for AD primarily offer symptomatic relief rather than halting disease progression [1,2]. Recent studies have suggested that dietary supplements with neuroprotective properties could serve as promising functional foods, particularly for elderly individuals seeking to prevent cognitive decline [1]. Numerous studies have demonstrated the potential benefits of dietary supplements, contributing to growing health awareness worldwide [1,3,4,5].

Among the various mechanisms contributing to age-related neurodegeneration, the formation of advanced glycation end products (AGEs) has gained considerable attention because of its role in exacerbating oxidative stress, neuroinflammation, and neuronal dysfunction [6,7]. Methylglyoxal (MGO), a highly reactive dicarbonyl compound derived from glycolytic intermediates, is a key AGE precursor [7,8]. Elevated MGO and AGE levels are associated with diabetes and aging, which are conditions that increase neuronal vulnerability and promote chronic neuroinflammation [7,8,9,10]. Consequently, strategies aimed at mitigating MGO-induced damage have emerged as potential interventions for the treatment of neurodegenerative diseases [8,11]. In our previous studies, MGO administration induced significant depression-like behaviors and memory deficits in animal models, which were accompanied by reduced levels of neurotransmitters (e.g., dopamine, epinephrine, and serotonin) and neurotrophic factors (e.g., as nerve growth factor and brain-derived neurotrophic factor) [7,10]. Notably, dietary supplementation with tryptophan alleviated these behavioral and cognitive impairments, suggesting its potential to modulate serotonin levels and promote neuroprotective mechanisms [12].

Building on these findings, we evaluated the neuroprotective efficacy of a combination therapy with trans-resveratrol (tRES) and hesperidin (HESP) in an MGO-induced neurodegeneration model. tRES, a polyphenolic compound abundant in grapes and peanuts, demonstrates robust antioxidant and anti-inflammatory properties, and protects neuronal cells from MGO-induced cytotoxicity [8,13]. Similarly, hesperidin (HESP), a flavonoid commonly found in citrus peels, exhibits neuroprotective effects by modulating oxidative stress and inflammatory pathways [14,15]. Previous studies have suggested that tRES significantly alleviates depressive symptoms and cognitive deficits in stroke models [16]. Its neuroprotective effects are mediated by the normalization of the hypothalamic-pituitary-adrenal (HPA) axis, restoration of neurotrophic support, and attenuation of inflammatory responses, indicating its therapeutic potential against both depression and cognitive impairment [16]. HESP administration ameliorates behavioral impairments by upregulating hippocampal neurotrophin expression and reducing pro-inflammatory cytokine levels, demonstrating its therapeutic promise for modulating neuroplasticity and synaptic function [14].

In addition to the mechanisms previously described, there is a growing body of evidence underscoring the beneficial impact of specific nutritional interventions on Alzheimer’s disease. Nutraceuticals, particularly trans-resveratrol and hesperidin, have emerged as promising compounds with potential positive influences on AD pathology. Trans-resveratrol, a polyphenolic phytoalexin abundantly present in grapes, berries, and peanuts, has been shown to mitigate the formation of beta-amyloid plaques and neurofibrillary tangles through its antioxidant and anti-inflammatory actions, partly via the activation of SIRT1 and modulation of key signaling pathways [17]. Similarly, hesperidin, as a flavonoid prevalent in citrus fruits, exerts neuroprotective effects by inhibiting Aβ aggregation, enhancing endogenous antioxidant defenses, and modulating inflammatory cascades through pathways such as RAGE/NFκB and Akt/Nrf2 [18]. Collectively, these compounds not only preserve mitochondrial function and promote neurogenesis, but also improve synaptic plasticity and cognitive performance. This evidence supports the inclusion of such nutraceuticals as adjunctive dietary supplements to alleviate neurodegenerative processes and promote brain health in individuals at risk for, or suffering from, AD. Although both tRES and HESP have been shown to exert anti-AD effects, their potential synergistic benefits in preventing MGO-mediated neuronal damage have not yet been fully elucidated [1,13,14,16]. This combination may provide a dual action approach to counteract MGO-induced neuronal damage and attenuate the progression of neurodegeneration. Therefore, the present study aimed to investigate whether the combined administration of trans-resveratrol and hesperidin (tRES-HESP) could mitigate MGO-induced neurotoxicity, improve cognitive deficits, and alleviate depressive symptoms using the ICR mouse model [12,14,16].

## 2. Materials and Methods

### 2.1. Materials

Dulbecco’s modified Eagle’s medium (DMEM), fetal bovine serum (FBS), MGO, aminoguanidine, donepezil, penicillin/streptomycin, tRES (180-02773; Wako, Richmond, VA, USA), HESP (PHR1794-500 mg; Merck Millipore, Burlington, MA, USA), dimethyl sulfoxide (DMSO), thiazolyl blue tetrazolium bromide (MTT), poly (ADP-ribose) polymerase (PARP) (9542s; Cell Signaling Technology, Danvers, MA, USA), BAX (2772s; Cell Signaling Technology), Bcl2 (3498s; Cell Signaling Technology), caspase 3 (9662s; Cell Signaling Technology), α-tubulin, glyoxalase 1 (sc-133214; Santa Cruz Biotechnology, Dallas, TX, USA), glyoxalase 2 (sc-166781; Santa Cruz Biotechnology), receptor for advanced glycation end-products (RAGE) (sc-365154; Santa Cruz Biotechnology), amyloid precursor protein (APP), mAβ MA5-36246; Invitrogen, Waltham, MA, USA), oAβ (AHB0052; Invitrogen), HO-1 (70081s; Cell Signaling Technology), Nrf2 (sc-365949; Santa Cruz Biotechnology), Kelch-like ECH-associated protein 1 (Keap1) (sc-514914, Santa Cruz Biotechnology), MTT, lactate dehydrogenase (LDH), BrDu, dichlorodihydrofluorescein diacetate (DCFH-DA), DAPI, interluekin (IL)-10, IL-1β, and cortisol.

### 2.2. Cell Viability

For the MTT assay, adherent neuroblastoma 2a (N2a) cells on a plate were exposed to MTT solution (0.5 mg/mL) for 1 h. The MTT solution was then aspirated and 100 μL of DMSO was subsequently added. Absorbance was recorded at 570 nm using a microplate reader (Bio-Rad, Hercules, CA, USA).

### 2.3. BrdU Cell Proliferation Assay

Cell proliferation was evaluated using the BrdU assay. N2a cells were plated at a density of 2 × 10^4^ cells per well in 96-well plates and incubated at 37 °C in an atmosphere containing 5% CO_2_ for 24 h. Subsequently, the cells were exposed to various concentrations of tRES and HESP and incubated in serum-free medium for 24 h. The effect of tRES+HESP on cell proliferation was quantified using a BrdU Cell Proliferation Assay Kit (#6813; Cell Signaling Technology).

### 2.4. LDH Assay

The LDH levels were determined using a cytotoxicity detection kit (Roche Diagnostics, Mannheim, Germany). Briefly, the enzymatic assay quantified LDH released from the cytosol of injured cells into the culture medium by converting the yellow tetrazolium salt INT (2-p-iodophenyl-3-p-nitrophenyl-5-phenyltetrazolium chloride) into a red formazan product.

### 2.5. Measurement of Intracellular Reactive Oxygen Species (ROS)

The ability of tRES-HESP to scavenge intracellular ROS was evaluated using the cell-permeable probe, DCFH-DA, which is hydrolyzed by intracellular esterases to form DCFH. In brief, 1 × 10^5^ cells were plated in a 35 mm confocal dish and incubated overnight at 37 °C. After 24 h, the cells were pre-treated with tRES-HESP for 1 h, followed by incubation with MGO for 2 h. Subsequently, the cells were washed with phosphate-buffered saline (PBS, pH7.4), and DAPI (1 µg/mL) was added. After a 10 min incubation at 37 °C, the cells were rinsed again with PBS. Following a final PBS wash, 20 µM DCFH-DA was introduced, and images were captured using a confocal fluorescence microscope (Nikon A1 Plus, Nikon, Tokyo, Japan). The fluorescence intensity of the images was quantified using the NIS-Elements software version 6.10.01.

### 2.6. Western Blot Analysis

N2a cells and brain tissues were homogenized in PRO-PREP™ protein extraction solution (iNtRON, Seoul, Republic of Korea) and maintained at −20 °C for 24 h. Following centrifugation, the protein concentration was measured using the Bradford assay. Protein aliquots, approximately 30–50 μg, were subjected to fractionation via sodium dodecyl sulfate-polyacrylamide gel electrophoresis (SDS-PAGE) and subsequently transferred onto polyvinylidene fluoride (PVDF) membranes employing a Trans-Blot^®^ Turbo™ transfer apparatus. The membranes were subjected to a blocking procedure with 5% skim milk solution for 1 h, prior to overnight incubation with primary antibodies at 4 °C. Following the nocturnal incubation period, the membranes were subjected to washing procedures and subsequently incubated with secondary antibodies for 1 h at ambient temperature (25 °C). The immunoreactive bands were ultimately visualized utilizing a ChemiDoc™ XRS+ imaging system (Bio-Rad) for densitometric analysis.

### 2.7. Animals and Study Design

**Figure 1:**
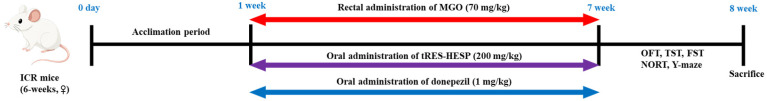
Schematic diagram of the experimental plan.

**Figure 2:**
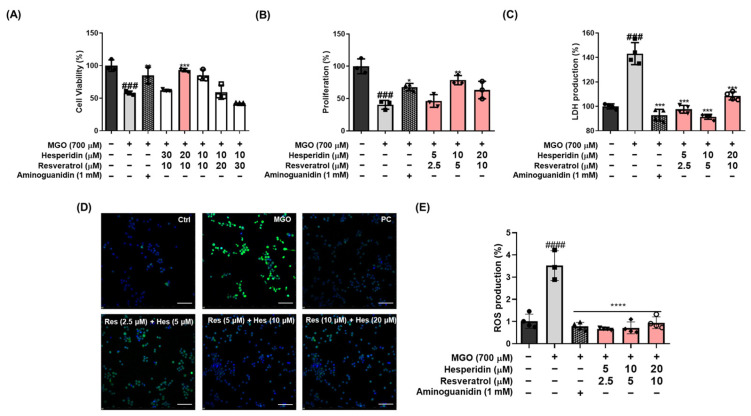
Cell viability (**A**), cell proliferation (BrdU assay, (**B**)), lactate dehydrogenase levels (**C**), and reactive oxygen species (ROS) levels (**D**,**E**) were measured on MGO-induced neuronal N2a cells. Scale bar: 100 μm (20× magnification). Ctrl, normal group; MGO, MGO-induced group; AG, 1 mM aminoguanidine (positive control) group. All data are presented as the mean ± SD (*n* = 3–4). ^###^ *p* < 0.001 and ^####^ *p* < 0.0001 vs. Ctrl, * *p* < 0.05, ** *p* < 0.01, *** *p* < 0.001, and **** *p* < 0.0001 vs. MGO-induced group (MGO).

ICR male mice (6 weeks old, *n* = 28, 37.07 ± 1.88 g) were obtained from Orient Bio (Seongnam-si, Republic of Korea) and housed under standardized laboratory conditions (22 ± 2 °C, 65% relative humidity, and a 12 h light/dark cycle). All animal procedures were approved by the Animal Care Committee of the Center for Animal Care and Use at Gachon University (Approval No. GU1-2022-IA0046). The mice were housed in polycarbonate cages (185 w × 340 d × 130 h mm) with aspen wood chip bedding that was sterilized at high temperature (131 °C) prior to use. The animals were housed with 3–4 mice per cage to maintain appropriate population density. Experimental subjects were allocated to four treatment groups using weight stratified randomization. Body weight measurements guided distribution to ensure homogeneous weight profiles across groups. All animals were incorporated into the study with no exclusions applied. After a one-week acclimatization period, mice were randomly assigned to four groups (*n* = 7 per group) as shown in Figure 1: (1) Control group as vehicle (CON), (2) MGO-treated group (70 mg/kg, MGO), (3) MGO + donepezil (1 mg/kg, positive control), and (4) MGO + tRES-HESP (200 mg/kg). MGO was administered via rectal injection using a 30% *v*/*v* glycerol/PBS solution (pH 7.4), while donepezil and tRES-HESP were administered orally once daily for eight weeks. Trans-resveratrol (tRES) and hesperidin (HESP) were sourced from Doctor’s Best (Tustin, CA, USA) and Nutrition Greenlife (Miami, FL, USA), respectively. Based on prior in vitro optimization (Figure 2A, Figures S1 and S2), the tRES-HESP mixture was prepared at a 1:2 ratio (tRES:HESP) for administration. The tRES-HESP was administered at a dosage of 200 mg/kg, according to previous studies that demonstrated improved neuroprotective effect [19,20]. The protocol involves exposing laboratory mice to CO_2_ in hermetically sealed containers. Verification of vital sign absence, including respiratory and cardiac functions, takes place after a minimum five minutes.

### 2.8. Open Field Test (OFT)

Following a methodology comparable to Ueno et al. with minor adjustments [18], the OFT was conducted utilizing a clear plastic apparatus (45 × 45 × 45 cm). Each apparatus had its interior compartmentalized into 24 equivalent squares (dimensions of 11.25 × 11.25 cm per square). Individual mice were positioned at the apparatus center and permitted unrestricted exploration, while their behavior was recorded on video for a duration of 5 min. The plastic apparatus underwent thorough sanitization with 70% ethanol between successive tests. Utilizing SMART3.0 SUPER PACK software V3.0.06 (Panlab; Harvard Apparatus, Barcelona, Spain), researchers analyzed various parameters including total distance traversed, duration spent, and velocity within the defined central region.

### 2.9. Novel Objective Recognition Test (NORT)

In several experiments, an acquisition trial was initially performed in which the mice were exposed to two identical objects. A probe trial succeeded the acquisition phase, wherein recognition memory was evaluated by substituting one of the familiar objects with a novel item. The testing environment and objects underwent meticulous cleansing between trials to eliminate olfactory traces from previously examined mice. The experimental design incorporated diverse plastic toys of varying configurations as test objects. Object exploration was operationally characterized as the commencement of sniffing behavior when the animal’s nose approached within a 1 cm proximity to the object; notably, behaviors such as climbing upon or perching atop the object were excluded from this definition. The assessment of recognition memory was accomplished through comparative analysis of exploratory durations directed toward both novel and familiar objects. The training trial lasted 5 min, after which the mice were returned to their home cages at 10 min intervals. Subsequently, the probe trial lasted for 3 min, during which one of the familiar objects was replaced with a novel object, and the time spent exploring each object was recorded [12].

### 2.10. Y-Maze Test

At one arm’s terminus of a symmetrical Y-maze apparatus, each experimental subject was positioned. The maze’s arms featured dimensions of 40 cm in length, 8 cm in width, and 20 cm in height. A 5 min interval was allotted during which the animals could navigate the maze without restriction. A ceiling-mounted camera documented the sequential pattern of arm entries (illustrated by sequences such as ACBCABCBCA). The criterion for identifying an alternation entailed the consecutive entry of a mouse into three different arms, forming overlapping triplet configurations (e.g., in the sequence ACBCABCBCA, five alternations were identified). The calculation of spontaneous alternation involved determining the proportion of actual alternations relative to the maximum possible number of alternations. As defined by previous methodology [12], this maximum value was computed by subtracting two from the total quantity of arm entries.

### 2.11. Tail Suspension Test (TST)

Implementation of the TST followed a modified version of the protocol established by Ueno et al. [18]. The experimental apparatus utilized was a chamber with dimensions of 60 cm (length) × 60 cm (height) × 11.5 cm (depth) × 15 cm (width). Animals were suspended via an adhesive tape-based fixation protocol specifically designed to minimize distress. A 2 min acclimation period was provided to each experimental subject prior to commencing data acquisition. Subsequently, individual mice were positioned within the apparatus and their behavioral responses were documented for a 4 min duration. Immobility parameters were subjected to quantitative assessment utilizing SMART3.0 SUPER PACK analytical software.

### 2.12. Forced Swimming Test (FST)

With slight procedural adjustments to the methodology outlined by Kang et al. [18], the FST was implemented. The experimental apparatus consisted of a cylindrical chamber (diameter of 20 cm and height of 50 cm) that contained water at ambient temperature (25 ± 1 °C) filled to a 30 cm depth. Each experimental subject underwent a 2 min habituation period within the chamber prior to data collection. Subsequently, mice were individually introduced into the apparatus, and their behavioral responses were documented for a 4 min interval. Quantitative analysis of immobility parameters was executed using SMART3.0 SUPER PACK analytical software.

### 2.13. Preparation of Mouse Plasma and Brain Tissue Sections

The preparation of plasma samples and brain tissue sections from mice followed protocols comparable to those documented in a previous publication [12]. Following euthanasia, cardiac puncture facilitated blood collection into EDTA-containing tubes to prevent coagulation. The plasma fraction was isolated after centrifugation (3000 rpm, 5 min) as the resultant supernatant. Transcardial perfusion was performed on the animals using 0.05 M PBS, with subsequent fixation implemented using 4% paraformaldehyde dissolved in 0.1 M phosphate buffer. Following extraction, the brain specimens underwent overnight post-fixation at 4 °C before being submerged in a cryoprotective 30% sucrose PBS solution. A microtome (Leica Microsystems Inc., Nussloch, Germany) was employed to section the brain tissues at a defined thickness of 25 μm for subsequent histological examination.

### 2.14. Enzyme-Linked Immunosorbent Assay (ELISA)

The pro-inflammatory cytokines (IL-6 and IL-10) and the stress-related cytokine cortisol in the MGO-induced models were quantified using ELISA kits (R&D Systems, Minneapolis, MN, USA). Serum samples were collected from mice after sacrifice, and cytokine levels were determined using appropriate kits according to the manufacturer’s protocol.

### 2.15. Hematoxylin and Eosin (H&E) Staining

Brain tissue was fixed in 10% formalin, dehydrated in ethanol, cleared in xylene, and embedded in paraffin blocks. The tissues were then sectioned at a thickness of 5 µm and stained using an H&E staining kit (Sigma-Aldrich, St. Louis, MO, USA). The stained slides were examined and photographed using an Eclipse 80i microscope (Nikon) at 4 and 10× magnification.

### 2.16. Immunochemistry (IHC) Analysis

The sections were obtained as described for the H&E assay, were deparaffinized using xylene, rehydrated with EtOH (100–70%), reacted with a peroxidase blocker, and washed with PBS. Each section was incubated with the (oAβ), monomeric Aβ (mAβ, Invitrogen, 1:100), and oligomeric Aβ (oAβ, Invitrogen, 1:100) antibodies at 4 °C overnight. After one day, the slides were incubated with biotinylated anti-rabbit IgG (dilution 1:200) for 1 h and then with an avidin-biotin horseradish peroxidase complex (Vector Laboratories, Newark, CA, USA). The optical density of mAβ and oAβ immunoreactivity in the hippocampus was measured using ImageJ 1.54k software (National Institutes of Health, Bethesda, MD, USA). Images were captured at 4 and 10× magnification using a microscope (Olympus, Tokyo, Japan).

### 2.17. Statistical Analysis

All results are expressed as the mean ± standard deviation (SD). All statistical analyses were performed using Prism 5.0 (GraphPad Software Inc., San Diego, CA, USA), SPSS software (version 25.0; IBM SPSS Statistics Inc., Chicago, IL, USA), and *t*-tests. Comparisons between the control and experimental groups were evaluated using the Bonferroni correction for multiple comparisons and one-way analysis of variance (ANOVA). Following a one-way ANOVA, Tukey’s post hoc test was used to determine the statistical significance of the cell viability assay and spine density analysis. Statistical significance was set at *p* < 0.05.

## 3. Results

### 3.1. Effect of tRES-HESP on MGO-Induced Cytotoxicity and ROS Production in N2a Cells

To investigate the potential protective effects of a tRES-HESP mixture against MGO-induced damage, N2a cells were pretreated with the mixture for 1 h prior to MGO (700 μM) exposure for 24 h. In this study, we used aminoguanidine as a positive control to inhibit glycotoxins. We initially evaluated tRES and HESP individually across a concentration range of 1 to 250 μM in MGO-treated N2a cells (Figure S1). The results indicated that cytotoxicity was observed at 25 μM (* *p* < 0.05) for tRES and 250 μM (* *p* < 0.05) for HESP. Therefore, we selected the highest nontoxic concentrations and determined the optimal mixture ratio to achieve synergistic effects (Figure 1). Notably, the tRES-HESP combination exhibited optimal synergy at a 1:2 ratio (tRES:HESP), yielding superior effects compared to the individual treatments (Figures S1 and S2). Based on these findings, we investigated the tRES-HESP mixture using the optimized ratio described above.

As shown in Figure 2A–C, the cells treated with MGO (^###^ *p* < 0.001) alone exhibited a significantly decreased loss of viability and proliferation and increased LDH production compared to the control group. However, treatment with tRES-HESP suppressed the loss of viability and proliferation in a concentration-dependent manner and significantly reduced LDH production.

Intracellular ROS levels were assessed using confocal imaging to observe the cytoprotective effects of tRES-HESP. Consistent with the MTT results, the MGO-treated group showed significantly elevated ROS production (^####^ *p* < 0.0001) compared to control cells. In contrast, tRES-HESP significantly reduced ROS generation in a dose-dependent manner. Notably, the highest concentration containing 10 μM tRES and 20 μM HESP (**** *p* < 0.0001) almost restored ROS levels to those observed in the untreated control group (Figure 2D,E).

### 3.2. Effects of tRES-HESP on MGO-Induced Expression of Related Apoptosis Pathways in N2a Cells

**Figure 3:**
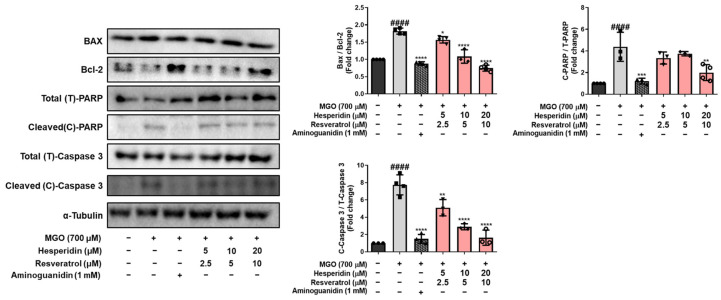
Western blotting to measure the expression of BAX/Bcl-2, cleaved (C)-PARP/total (T)-PARP, and cleaved (C)-caspase-3/total (T)-caspase-3 in MGO-induced N2a cells. All protein levels were normalized to those of α-tubulin. All data are presented as the mean ± SD (*n* = 3–4). ^####^ *p* < 0.0001 vs. Ctrl, * *p* < 0.05, ** *p* < 0.01, *** *p* < 0.001, and **** *p* < 0.0001 vs. MGO-induced group (MGO).

We further found that treatment with MGO (700 μM) for 24 h significantly increased the expression of pro-apoptotic markers, including BAX and cleaved caspase-3, while decreasing the anti-apoptotic protein Bcl-2, compared with untreated control cells. In contrast, treatment with tRES-HESP for 1 h reversed these effects in a concentration-dependent manner (Figure 3). Specifically, at 15 and 30 μM of tRES-HESP, the levels of BAX (^####^ *p* < 0.0001) and cleaved caspase-3 (^####^ *p* < 0.0001) were dramatically suppressed, whereas those of Bcl-2 (^####^ *p* < 0.0001) were significantly restored toward control levels. Furthermore, cleavage of PARP, another indicator of apoptosis, was markedly reduced following treatment with the mixture, suggesting the inhibition of the apoptotic cascade. In particular, treatment tRES-HESP at high concentration (30 μM) showed an almost complete reduction of cleaved caspase-3 (**** *p* < 0.0001) and cleaved PARP (** *p* < 0.01), with levels similar to those in the positive control group (AG). These results indicated that tRES-HESP suppressed MGO-induced neuronal apoptosis by modulating the expression of key apoptotic regulators, thereby exerting a potent cytoprotective effect in N2a cells.

### 3.3. Effects of tRES-HESP on the MGO-Induced Glyoxalase System in N2a Cells

**Figure 4:**
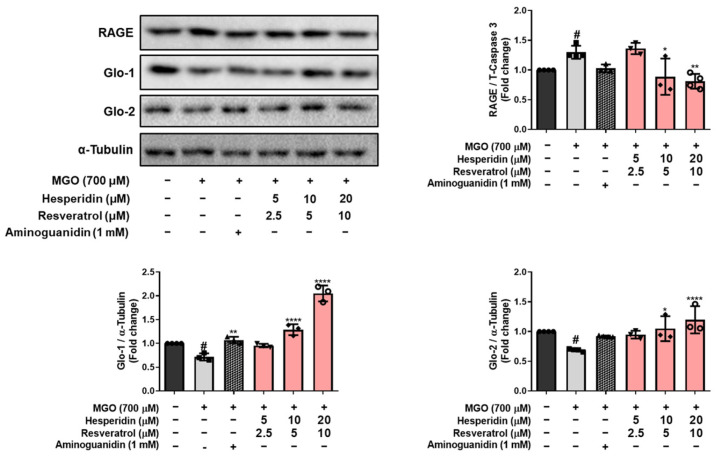
Western blotting to measure the expression of RAGE, Glo-1, and Glo-2 in MGO-induced N2a cells. All protein levels were normalized to those of α-tubulin. All data are presented as the mean ± SD (*n* = 3–4). ^#^ *p* < 0.05 vs. Ctrl, * *p* < 0.05, ** *p* < 0.01, and **** *p* < 0.0001 vs. MGO-induced group (MGO).

To gain insight into the underlying protective mechanisms of tRES-HESP against MGO-induced damage in N2a cells, we examined the expression of RAGE, glyoxalase-1 (Glo-1), and glyoxalase-2 (Glo-2). As shown in Figure 4, exposure to MGO alone significantly decreased the expression of Glo-1 (^#^ *p* < 0.05) and Glo-2 (^#^ *p* < 0.05), while concomitantly upregulating RAGE (^#^ *p* < 0.05), compared to the control group. In contrast, treatment with tRES-HESP resulted in pronounced concentration-dependent restoration of Glo-1 and Glo-2 expression, accompanied by significant downregulation of RAGE levels. In particular, treatment with 30 μM tRES-HESP markedly enhanced Glo-1 (**** *p* < 0.0001) and Glo-2 (**** *p* < 0.0001) expression compared to that under MGO-only conditions, reaching levels comparable to those of the control group. RAGE expression (** *p* < 0.01) was also dramatically reduced with tRES-HESP treatment at 30 μM, showing an approximately 40% decrease relative to MGO-treated cells. These results suggest that the glyoxalase system, along with RAGE modulation, plays an essential role in the anti-glycotoxic and cytoprotective effects of resveratrol and hesperidin in MGO-stressed neurons.

### 3.4. Effects of tRES-HESP on MGO-Induced Antioxidant Defense Mechanisms in N2a Cells

**Figure 5:**
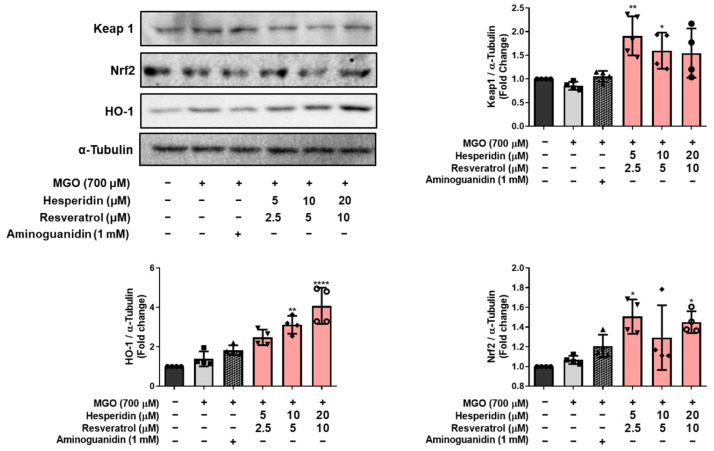
Western blotting assay to measure the expression of Keap1, Nrf2, and HO-1 in MGO-induced N2a cells. All protein levels were normalized to those of α-Tubulin. All data are presented as the mean ± SD (*n* = 4). * *p* < 0.05, ** *p* < 0.01 and **** *p* < 0.0001 vs. MGO-induced group (MGO).

Having observed that tRES-HESP modulates the glyoxalase system in MGO-stressed N2a cells, we next examined changes in the Keap1/Nrf2/HO-1 antioxidant pathway to better understand the underlying protective mechanisms. As shown in Figure 5, MGO treatment alone significantly increased the expression levels of Keap1 and decreased both Nrf2 and HO-1 expression compared to those in the control group. However, treatment with tRES-HESP reversed this effect in a concentration-dependent manner. Specifically, cells exposed to tRES-HESP showed a marked reduction in Keap1 (**** *p* < 0.0001) and corresponding upregulation of Nrf2 and HO-1. This alteration in the expression profile indicated that tRES-HESP counteracted MGO-induced suppression of the endogenous antioxidant defense system by stabilizing Nrf2, thereby facilitating the upregulation of HO-1 expression. These data indicate that MGO-induced oxidative stress is mitigated by tRES-HESP co-treatment via the Keap1/Nrf2/HO-1 axis, further supporting the potent cytoprotective role of this mixture against MGO-induced neuronal damage.

### 3.5. The Treatment of tRES-HESP Alleviates MGO-Induced Depression-like Behavior and Cognitive Deficits in Mice Models

**Figure 6:**
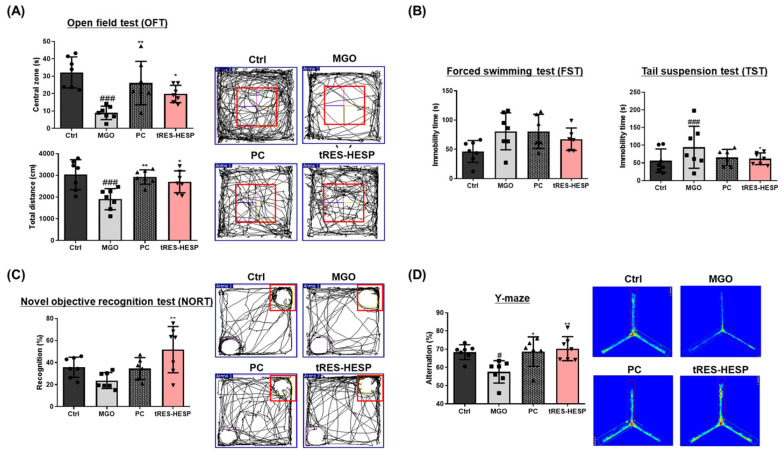
(**A**) The time spent and total distance in the open field test (OFT). (**B**) Immobility time in the forced swimming test (FST) chamber and tail suspension test (TST) chamber. (**C**) The recognition percentage in novel object tool and total distance in the novel objective recognition test (NORT). (**D**) The alternation percentage and total distance in the Y-maze test. Each behavior test was analyzed using SMART3.0 SUPER PACK (Panlab; Harvard Apparatus, Barcelona, Spain). All data are presented as the mean ± SD (*n* = 6–7). ^#^ *p* < 0.01 and ^###^ *p* < 0.001 vs. Ctrl, * *p* < 0.05, ** *p* < 0.01 vs. MGO-induced group (MGO).

To determine whether MGO-induced depression-like behaviors could be alleviated by treatment with tRES-HESP, we administered tRES-HESP (200 mg/kg) for 8 weeks to ICR mice in the presence or absence of MGO (Figure 6A–C). In the open field test (OFT), MGO treatment significantly reduced the time spent in the central zone (19.80 ± 1.872 s, * *p* < 0.05) compared to the control group. The red box indicates the central zone, where increased time spent reflects reduced anxiety levels in subjects. Notably, this parameter was restored to control levels in the tRES-HESP group, indicating an anxiolytic-like effect (Figure 6A). MGO-treated groups showed prolonged immobility times (a hallmark of depression-like behavior) in both the FST (55.55 ± 5.923 s, ** *p* < 0.01) and TST (67.42 ± 7.255 s, * *p* < 0.05) (Figure 6B). However, the tRES-HESP group showed significantly decreased immobility times, suggesting that treatment with tRES-HESP mitigated MGO-induced depressive symptoms. Treatment with donepezil (1 mg/kg, PC), a potent acetylcholinesterase inhibitor, also resulted in improvements, albeit to a lesser extent than in the tRES-HESP group. Collectively, these findings indicated that tRES-HESP confers robust protection against MGO-induced depression-like behaviors in mice.

### 3.6. Protective Effects of tRES-HESP on MGO-Induced Cognitive Dysfunction

To determine whether MGO-induced memory deficits could be ameliorated by tRES-HESP, we performed the NORT and Y-maze tests (see Figure 6C,D). In the NORT, the red box indicates novel object placement. Increased exploration time in this zone reflects enhanced recognition memory in subjects. In the NORT, MGO-induced mice (23.36 ± 2.70%) exhibited a significantly reduced recognition percentage compared to controls, but this reduction was reversed by tRES-HESP treatment (54.15 ± 10.48%) (Figure 6C). Similarly, MGO (55.40 ± 2.03%, ^##^ *p* < 0.01) exposure decreased the alternate percentage in the Y-maze, indicative of impaired spatial working memory; however, the treated tRES-HESP (73.23 ± 3.63%, ** *p* < 0.01) restored this parameter toward control levels (Figure 6D). Notably, the total number of arm entries remained comparable across the groups, suggesting that general locomotor activity was unaffected. These findings imply that tRES-HESP effectively mitigated MGO-induced memory loss in mice.

### 3.7. Effects of tRES-HESP on Cytokines and Cortisol in Mouse Serum

**Figure 7:**
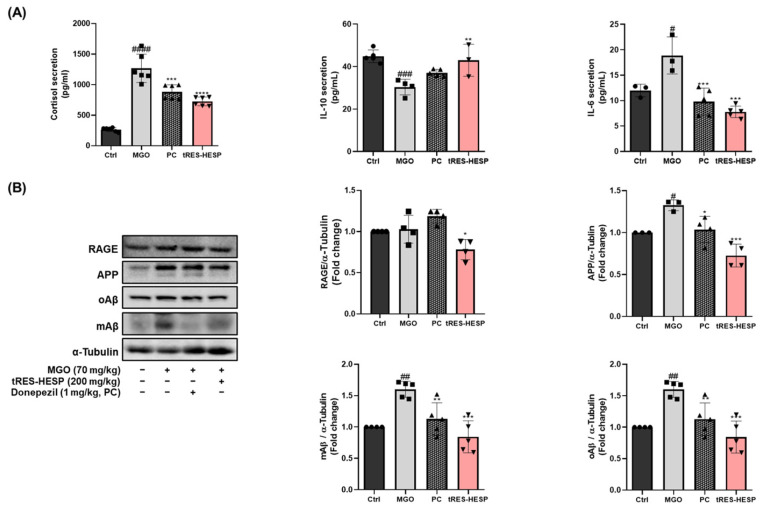
(**A**) The secreted levels of interleukin (IL)-6, IL-10, and cortisol in the serum measured using ELISA assay kits. (**B**) Western blotting to measure the expression of RAGE, APP, oAβ, and mAβ. All protein levels were normalized to those of α-tubulin. All data are presented as the mean ± SD (*n* = 3–6). ^#^ *p* < 0.05, ^##^ *p* < 0.01, ^###^ *p* < 0.001 and ^####^ *p* < 0.0001 vs. Ctrl, * *p* < 0.05, ** *p* < 0.01, *** *p* < 0.001, and **** *p* < 0.0001 vs. MGO-induced group (MGO).

To assess the impact of tRES-HESP on MGO-induced changes in cytokine and stress hormone levels, we performed ELISAs for IL-10, IL-6, and cortisol (Figure 7A). The MGO-treated group showed significantly reduced IL-10 (30.35 ± 3.63 pg/mL, ^####^ *p* < 0.0001) and elevated IL-6 (18.87 ± 1.12 pg/mL, ^#^ *p* < 0.05) and cortisol (1266.29 ± 80.22 pg/mL, ^####^ *p* < 0.0001) secretion compared with the control group. Notably, the treated tRES-HESP group significantly reversed these alterations (IL-10, 42.94 ± 2.32 pg/mL; IL-6, 7.78 ± 0.39 pg/mL; and cortisol, 726.15 ± 24.70 pg/mL). Similar trends were observed in the donepezil-treated group (IL-10, 40.27 ± 1.80 pg/mL; IL-6, 9.80 ± 0.90 pg/mL; and cortisol, 880.15 ± 40.56 pg/mL). Collectively, these results suggest that cotreatment with tRES-HESP modulated inflammatory and stress responses in MGO-challenged mice by enhancing anti-inflammatory cytokine secretion and reducing pro-inflammatory cytokine and cortisol levels.

### 3.8. Effects of tRES-HESP on MGO-Induced Amyloid Pathology in the Mice Brain

Mice were treated with tRES-HESP and injected with MGO to induce neurodegenerative changes in the brain, and the expression of amyloid-related proteins was examined. The MGO-treated group showed a marked increase in APP (^#^ *p* < 0.05), oligomeric Aβ (oAβ, (^##^ *p* < 0.01), and monomeric Aβ (mAβ, ^##^ *p* < 0.01) levels, as well as a significant elevation in RAGE compared to the control group (Figure 7B). In particular, densitometric analysis revealed that the MGO group exhibited an approximately 2–3-fold rise in APP and Aβ species relative to the control group. Conversely, the tRES-HESP group exhibited significantly reduced expression of APP (*** *p* < 0.001), oAβ (*** *p* < 0.001), and mAβ (*** *p* < 0.001), with reduction of approximately 30–40% compared to the MGO-only group. Moreover, RAGE expression was substantially downregulated by tRES-HESP treatment (* *p* < 0.05), indicating that the mixture effectively inhibited the glycation-driven amyloidogenic pathway. Indeed, the IHC results further supported that tRES-HESP effectively inhibited the amyloid pathology (Figure 7). Taken together, these findings suggest that treatment with tRES-HESP attenuates MGO-induced amyloid pathology, potentially contributing to improved neuronal homeostasis in this neurodegenerative model.

### 3.9. tRES-HESP Mitigates MGO-Induced Amyloid Deposition and Neuronal Damage in the Hippocampus

**Figure 8:**
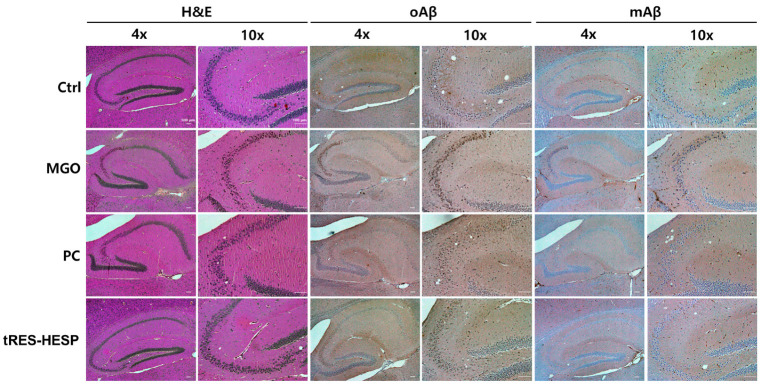
Histological analysis of the CA3 region in the hippocampus. Representative images of H&E staining and immunohistochemical (IHC) staining for oligomeric Aβ (oAβ) and monomeric Aβ (mAβ) in the CA3 regions of the hippocampus. Scale bars: 100 μm (4× and 10× magnification).

To evaluate histopathological changes and amyloid deposition, mouse brains were sectioned and subjected to IHC for mAβ and oAβ, as well as H&E staining to assess neuronal integrity in the hippocampus. In the MGO group, IHC revealed a pronounced accumulation of both mAβ and oAβ in the CA3 subregion of the hippocampus, indicative of extensive amyloid deposition (Figure 8). H&E staining corroborated these findings by showing marked neuronal damage characterized by cellular shrinkage, loss of structural integrity, and disrupted cytoarchitecture in the CA3 region, suggesting significant MGO-induced hippocampal injury (Figure 8). In contrast, the PC and tRES-HESP groups exhibited significantly reduced Aβ immunoreactivity, and H&E staining demonstrated preserved neuronal morphology with minimal histopathological alterations in the CA3 region. These results support the neuroprotective effects of tRES-HESP in mitigating MGO-induced amyloid pathology and hippocampal neuronal damage.

## 4. Discussion

In the present study, we investigated the neuroprotective potential of a combined treatment with tRES-HESP against MGO-induced neurotoxicity using both in vitro and in vivo models. MGO, a dicarbonyl compound, reacts with proteins, lipids, and DNA, producing AGEs that induce cellular damage [21]. MGO plays a crucial role in the pathogenesis of neurodegenerative diseases including AD [22]. Several reports have revealed that MGO contributes to neurodegeneration via oxidative stress, such as ROS, and induces apoptosis in hippocampal neurons by activating numerous intracellular signaling pathways [23]. Moreover, our previous study found that MGO reduced the number of cells in the hippocampus formation, such as cornu ammonis 1 (CA1), CA3, and dentate gyrus (DG) and induced the accumulation of APP and Aβ to increase depression-like and cognitive impairment behaviors. The levels of pro-inflammatory cytokines (e.g., IL-1β, IL-6, and TNF-α) and Iba-1 were also increased through microglial activation [12]. Given that MGO, as an endogenous factor, appears to contribute to brain damage, we hypothesized that elevated MGO levels might enhance oxidative stress and inflammation, thereby leading to depression-like symptoms and memory impairments. Furthermore, our MGO-induced mouse model holds promise as a valuable platform to evaluate the regulatory effects of dietary supplements and therapeutic agents. Interestingly, our findings demonstrate that the tRES-HESP significantly mitigates MGO-induced neuronal damage by attenuating oxidative stress, apoptosis, and glycation-mediated dysregulation.

The intrinsic apoptotic pathway contributes to programmed cell death induced by oxidative stress [24]. Neuronal apoptosis plays a pivotal role in the pathogenesis of neurodegenerative diseases, including AD. In this study, we evaluated whether tRES-HESP, a combination of antioxidants, protects against MGO-induced apoptosis and oxidative stress. In MGO-treated N2a neuronal cells, tRES-HESP markedly restored cell viability and reduced excess ROS production (Figure 2). In addition, tRES-HESP treatment increased cell proliferation and suppressed LDH production. Bax and Bcl-2 proteins modulate cell death, and caspases including caspase-3 are important regulators of apoptosis that respond to apoptotic stimuli [25]. The cytoprotective effects of tRES-HESP treatment was accompanied by the inhibition of key apoptotic markers, including Bax, cleaved caspase-3, and cleaved PARP as a caspase-3 nuclear substrate (Figure 3). Therefore, tRES-HESP appeared to play a crucial role in MGO-induced apoptosis in N2a cells via intrinsic apoptotic pathways involving Bax/Bcl-2, PARP, and caspase-3.

MGO is detoxified into d-lactate via the glyoxalase system by Glo-1 and Glo-2 enzymes, which convert D-lactate into S-D-lactoylglutathione via glutathione-dependent pathways [26]. tRES-HESP treatment effectively reversed the MGO-induced suppression of the glyoxalase system by upregulating Glo-1 and Glo-2, indicating that tRES-HESP may support the glyoxalase systems to detoxify MGO. Moreover, MGO induces AGE formation, which binds to RAGE and initiates neurodegenerative diseases such as AD [6]. Our results showed that tRES-HESP treatment downregulated RAGE, which was observed to increase after treatment with MGO (Figure 4 and Figure 7B). These findings suggest that tRES-HESP alleviates glycation-mediated cytotoxicity by restoring detoxification mechanisms and inhibiting the formation of MGO-derived AGEs, which binds to RAGE.

Additionally, our data indicate that tRES-HESP modulates the cellular antioxidant defense system. Elevated MGO levels in neuronal cells activate the Nrf2 signaling pathway, leading to cytoprotective effects through the upregulation of HO-1 and modulation of Keap1 [27]. MGO treatment resulted in increased expression of Keap1 and decreased levels of Nrf2 and HO-1, thereby impairing endogenous antioxidant capacity, but tRES-HESP treatment reversed these alterations in a concentration-dependent manner. It suggests that the activation of the Keap1/Nrf2/HO-1 pathway plays a critical role in counteracting MGO-induced oxidative stress (Figure 5).

Behavioral assessments in MGO-challenged mice further supported the neuroprotective effects observed in vitro. Therefore, we administered tRES-HESP to MGO-treated mice to investigate its role in the brain. We found that tRES-HESP administration significantly improved the time spent in the central area in the OFT, and the baseline immobility time of the mice decreased (Figure 6A,B). In addition, treatment with tRES-HESP significantly enhanced the recognition index in the NORT, indicating improved spatial memory in mice (Figure 6C). Similarly, the percentage of spontaneous alternations in the Y-maze test also increased (Figure 6D). Thus, the results of each behavior suggested the efficiency of tRES-HESP in rescuing brain dysfunction. In addition, detailed histopathological analyses revealed that the CA2 and CA3 subregions of the hippocampus were particularly susceptible to MGO-induced damage. Specifically, MGO exposure led to significant morphological disruptions, a decline in synaptic marker expression, and an increase in oxidative stress and apoptotic signaling within these subfields. Notably, the administration of tRES-HESP markedly attenuated these deleterious effects, restoring both the structural integrity and functional capacity of the CA2 and CA3 regions (Figure 8). These observations align with those of previous studies, indicating that the CA2–CA3 subfields are highly vulnerable to oxidative and glycation stress [20,21].

Interestingly, our results showed that tRES-HESP suppressed amyloidogenesis in the brain. It is well-established that Aβ peptides are major markers associated with the pathogenesis of AD [12]. Moreover, MGO-derived AGEs have been shown to upregulate APP and Aβ expression, thereby contributing to various memory deficits associated with brain dysfunction [12], indicating that tRES-HESP may mitigate amyloid-related neurotoxicity, thereby preserving neuronal function. Moreover, we observed that tRES-HESP significantly reduced elevated cortisol levels in an MGO-induced mouse model. The dysregulation of the HPA axis by MGO-induced stress has been associated with anxiety, depression, and memory dysfunction [12]. Within the HPA axis, stress hormones including cortisol are upregulated in response to excessive immune activation and inflammation. These findings suggest that the MGO-induced elevation in cortisol, a key factor contributing to depressive symptoms, may be mitigated by tRES-HESP treatment via modulation of the HPA axis.

Additionally, MGO persistently activated the mitogen-activated protein kinase (MAPK) family and the nuclear factor kappa B (NF-κB) pathway, which may be associated with the enhancement of amyloidogenic processes and neuroinflammation [12]. In parallel, tRES-HESP normalized the serum levels of pro-inflammatory cytokines (Figure 7A), including IL-6, IL-10, and cortisol, and reduced the expression of amyloid-related proteins in the brain, suggesting a potential attenuation of both neuroinflammation and amyloidogenic pathways.

Despite promising preclinical results demonstrating the neuroprotective effects of tRES-HESP, translating these findings to clinical applications faces significant challenges. Species-specific differences in drug metabolism, pharmacokinetics, and the multifactorial nature of AD make it difficult to directly extrapolate optimal dosing from animal models to humans. Additionally, although the combination appears to exert synergistic neuroprotective effects, the precise mechanisms of interaction, whether additive or truly potentiated, remain unclear. Addressing these uncertainties through targeted pharmacodynamic studies and early-phase clinical trials is essential to optimize dosing strategies, ensure safety, and fully realize the therapeutic potential of this combination in treating neurodegenerative disorders.

## 5. Conclusions

This study provides compelling evidence that the combination of trans-resveratrol and hesperidin (tRES-HESP) offers potent neuroprotection against methylglyoxal (MGO)-induced toxicity. Through both in vitro and in vivo models, tRES-HESP was shown to restore neuronal viability, reduce oxidative stress and apoptosis, modulate inflammatory and glycation pathways, and improve cognitive and emotional behaviors. These effects appear to be mediated by the regulation of the glyoxalase system, RAGE expression, and the Keap1/Nrf2/HO 1 antioxidant pathway. Importantly, tRES-HESP also attenuated amyloid pathology and preserved hippocampal integrity, highlighting its relevance to AD models. Taken together, these findings suggest that tRES-HESP may serve as a promising dietary intervention for the prevention or mitigation of neurodegenerative disorders. Further clinical studies are necessary to confirm its efficacy and establish optimal dosing in human populations.

## Data Availability

All data generated or analyzed in this study are included in this article and its Supplementary Data Files.

## Supplementary Materials

The following supporting information can be downloaded at: https://www.mdpi.com/article/10.3390/nu17091548/s1. Figure S1. Effects of trans-resveratrol (tRES) and hesperidin (HESP) on MGO-induced cytotoxicity in N2a cells. (A) Cell viability of tRES (only). (B) Cell viability of tRES on MGO-induced cytotoxicity in N2a cells. (C) Cell viability of HESP (only). (D) Cell viability of HESP on MGO-induced cytotoxicity in N2a cells. ^###^ *p* < 0.001 vs. Ctrl, * *p* < 0.05, ** *p* < 0.01, or *** *p* < 0.001 vs. MGO-induced group (MGO). Figure S2. Effects of the mixture of trans-resveratrol and hesperidin (tRES+HESP) at various mixture ratios on MGO-induced cytotoxicity in N2a cells. (A) Cell viability of tRES+HESP (only). (B) Cell viability of tRES+HESP at various ratios on MGO-induced cytotoxicity in N2a cells. (C) Representative photographs of tRES+HESP at 1:2 ratio (10 µM of tRES and 10 µM of HESP) pre-treatment protection against MGO-induced cytotoxicity. Scale bar indicates 500 μm.
