# Supplementary material for: Neuroprotective Effects of a Combination of Dietary Trans-Resveratrol and Hesperidin Against Methylglyoxal-Induced Neurotoxicity in a Depressive Amnesia Mouse Model
Source: Nutrients. 2025 Apr 30;17(9):1548. doi: 10.3390/nu17091548 (PMC12074085; doi:10.3390/nu17091548)
Supplement: Supplementary file 1 [file nutrients-17-01548-s001.zip › nutrients-3592230-supplementary.pdf]

<Supplementary data>

Title: Neuroprotective effects of a combination of dietary trans-resveratrol and hesperidin against methylglyoxal -induced neurotoxicity in a depressive amnesia mouse model

<Supplementary methods>

Cell viability or and morphological changes

N2a cells were plated at a density of 2 × 104 cells per well in a 96-well plates and incubat-ed at 37 °C in an atmosphere containing 5% CO2 for 24 h. Subsequently, the cells were ex-posed to various concentrations of trans-resveratrol (tRES), hesperidin (HESP), or mixed each compound (tRES+HESP) and incubated in serum-free medium for 24 h. Adherent neuroblastoma 2a (N2a) cells on a plate were exposed to MTT solution (0.5 mg/mL) for 1 h. The MTT solution was then aspirated and 100 μL of DMSO was subsequently added. Absorbance was recorded at 570 nm using a microplate reader (Bio-Rad, Hercules, CA, USA). Cell morphological changes in mesangial cells were investigated using the IncuCyte Zoom imaging system (Essen Bioscience, Ann Arbor, MI, USA).

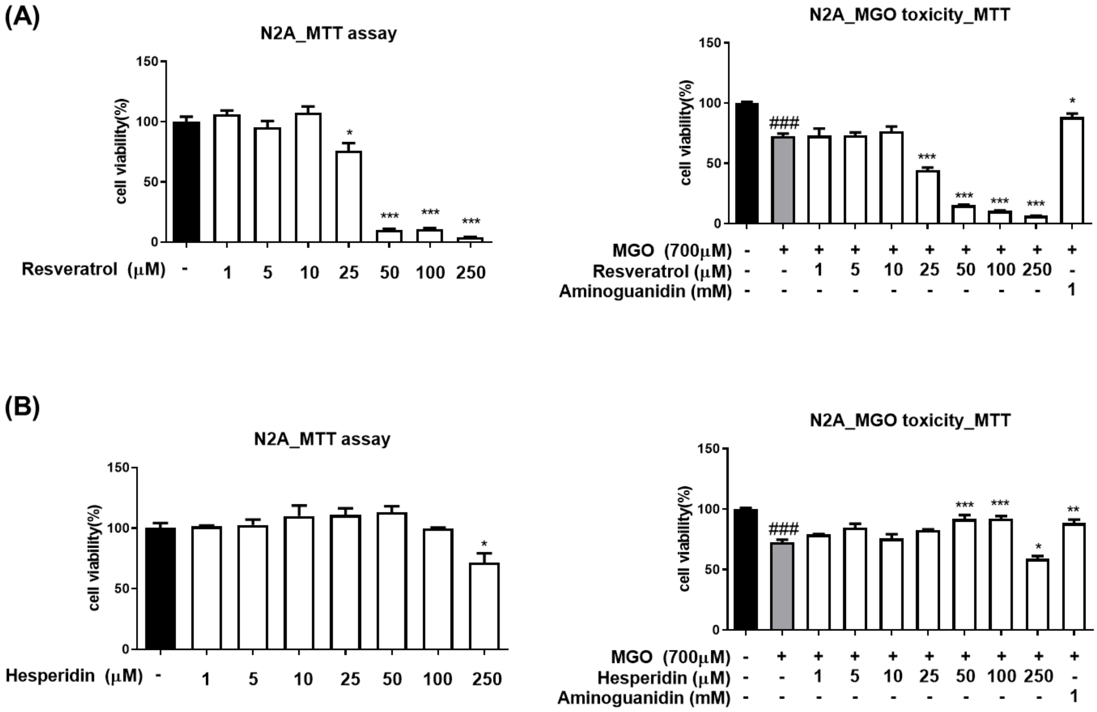

Figure S1. Effects of trans-resveratrol (tRES) and hesperidin (HESP) on MGO-induced cytotoxicity in N2a cells. (A) Cell viability of tRES (only). (B) Cell viability of tRES on MGO-induced cytotoxicity in N2a cells. (C) Cell viability of HESP (only). (D) Cell viability of HESP on MGO-induced cytotoxicity in N2a cells. $^{###}P < 0.001$ vs. Ctrl, $^{*}P < 0.05$, $^{**}P < 0.01$, or $^{***}P < 0.001$ vs. MGO-induced group (MGO).

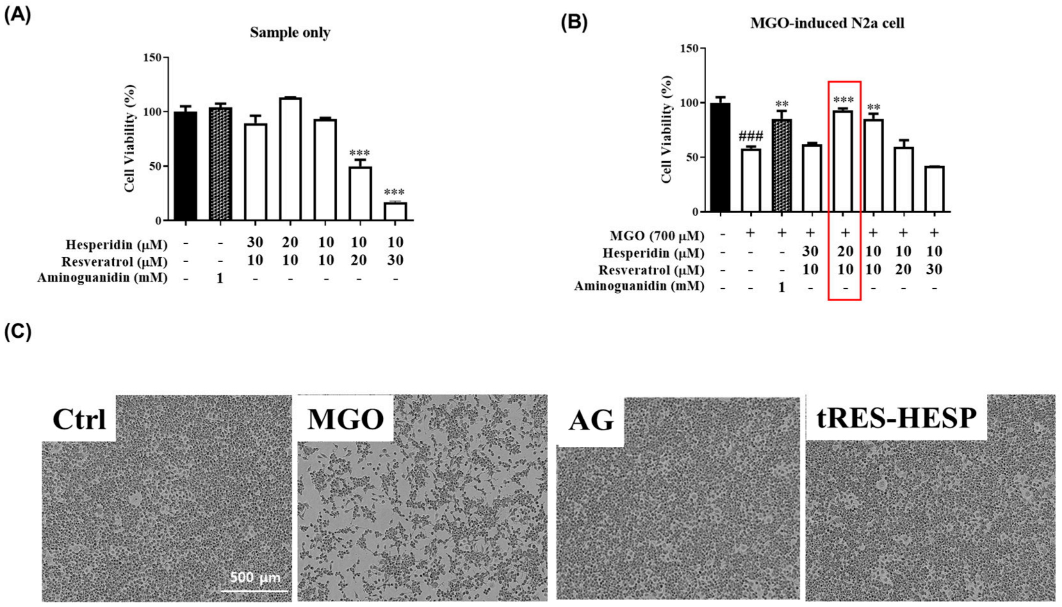

Figure S2. Effects of the mixture of trans-resveratrol and hesperidin (tRES+HESP) at various mixeture ration on MGO-induced cytotoxicity in N2a cells. (A) Cell viability of tRES+HESP (only). (B) Cell viability of tRES+HESP at various ratio on MGO-induced cytotoxicity in N2a cells. (C) Representative photographs of tRES+HESP at 1:2 ratio (10 μM of tRES and 10 μM of HESP) pre-treatment protection against MGO-induced cytotoxicity. Scale bar indicates 500 μm.
